# The role of nurses to control beta thalassemia disease in Indonesia: A perspective

**DOI:** 10.1016/j.jtumed.2023.02.007

**Published:** 2023-02-20

**Authors:** Henri Setiawan, Andan Firmansyah, Selvia D. Richard

**Affiliations:** a Department of Nursing, STIKes Muhammadiyah Ciamis, Indonesia; b Department of Nursing, STIKes RS Baptis Kediri, Indonesia

**Keywords:** Beta thalassemia, Community nurses, Preventive, Promotive

## Abstract

Thalassemia is the most common genetic disease in Indonesia and is passed on to the next generation following an autosomal recessive Mendelian inheritance pattern. The number of thalassemia sufferers in Indonesia increased from 4896 in 2012 to 8761 in 2018. The latest data in 2019 shows a significant increase to 10,500 patients. Community nurses who work at the Public Health Center, have full roles and responsibilities in carrying out promotive and preventive efforts against thalassemia cases. Promotive efforts that can be carried out are guided by government policies (Ministry of Health, Republic of Indonesia) which stipulate primary efforts in the form of education about thalassemia disease, prevention efforts, and diagnostic tests that can be taken. To optimize the promotive and preventive efforts, community nurses need to collaborate with midwives and cadres at integrated service posts. Interprofessional collaboration between stakeholders can strengthen the government's consideration in making policies for dealing with thalassemia cases in Indonesia.

## Dear Editor,

Thalassemia is the most common of inherited genetic disease in Indonesia and is passed on to the next generation following an autosomal recessive Mendelian inheritance pattern. The number of thalassemia sufferers in Indonesia increased from 4896 in 2012 to 8761 in 2018. The latest data in 2019 shows a significant increase to 10,500 patients. This data is predicted to continue to grow with as many as 1500 new cases diagnosed each year.^1^ An identified genetic disorder caused by mutations on chromosome 11 and chromosome 16.^2^ This mutation is the cause of the loss of the β-globin gene (the cause of β-thalassemia) and the α-globin gene (the cause of α-thalassemia). Genetically, this mutation causes a decrease or loss of β-globin chain synthesis, which is the largest component of adult hemoglobin. This situation directly affects the physical health of the patient and indirectly impacts the psychosocial status of the patient, parents (caregiver) and family.^1^ Anemia is a characteristic of thalassemia sufferers impact to depend on routine blood transfusions for their whole life. Other clinical manifestations that appear are splenomegaly, short stature, pale, and fatigue which have an impact on limiting the patient's activities. Depression, anxiety, worry and quality of life are psychosocial problems that are often reported in sufferers and families with thalassemia.^3^

Apart from consanguinity or marriage in the same family, awareness of carrying out carrier screening in hospitals and laboratories is still very low. Even families who have children with thalassemia do not do genetic testing. The most compelling reason given by the family was that the examination fee was expensive and not covered by health insurance.^4^ As a result, carrier tracking cannot be carried out, so it is possible for inter-carrier marriages to occur which have the potential to pass on 25% of offspring with thalassemia disease and 50% of new carriers. This bad potential must be prevented by strengthening the role and function of health workers, especially community nurses.

Community nurses who work at the Public Health Center, have full roles and responsibilities in carrying out promotive and preventive efforts against thalassemia cases. Promotive efforts that can be carried out are guided by government policies (Ministry of Health, Republic of Indonesia) which stipulate primary efforts in the form of education about thalassemia disease, prevention efforts, and diagnostic tests that can be taken. The hope is that the public will have early awareness of thalassemia disease.^5^

The preventive efforts that can be made are screening, tracing, and genetic counseling. Thalassemia screening can be done in all public hospitals by examining MCV (mean corpuscular volume), MCH (mean corpuscular hemoglobin), and MCHC (mean corpuscular hemoglobin concentration). To be more sure, further examination can be done with electrophoresis in hospitals and more complete laboratories.^6^ This screening is very important, especially for couples who are about to get married (premarital screening), so nurses need to collaborate with the Office of Religious Affairs, which has authority over the marriage process. Genetic counseling is given before and after carrier screening is taken, so that individual couples can make choices after getting strong information regarding the results of the examination. Although the nurse as a counselor does not have the authority to make choices, a detailed explanation regarding the risks arising from inter-carrier thalassemia marriage can be a strong consideration for couples.^7^

Tracing begins with drawing a family pedigree on families who have children with thalassemia or microcytic anemia. The description of family pedigree involves at least three generations in one family, so identification is taken comprehensively, including planning genetic counseling to be carried out. Parents who have children with Thalassemia should be suspected of being a carrier partner.^2^ Likewise, with family members in one pedigree, there may be carrier thalassemia. However, this allegation still needs to be proven by further investigations such as electrophoresis, because there are other possible causes of mutations (besides being inherited from a carrier partner), such as radiation, infection, toxicity, pollution, and even de novo. The results of this examination will be taken into consideration by parents in planning their next pregnancy. For other family members who are diagnosed as carriers based on the results of the examination, it is necessary to plan premarital screening for couples who are getting married.^2^

To optimize the promotive and preventive efforts, community nurses need to collaborate with midwives and cadres at integrated service posts. In addition to playing a role in obstetrical health checks, which are the main requirements before marriage, midwives can improve information about thalassemia, the importance of carrier screening, and emotional support.^8^ The cadres play an important role in providing assistance to families and communities diagnosed with carriers, so that awareness grows to participate in screening, tracing, and genetic counseling programs in public health centers and hospitals. Interprofessional collaboration between stakeholders can strengthen the government's consideration in making policies for dealing with thalassemia cases in Indonesia.

## Source of funding

This research did not receive any specific grant from funding agencies in the public, commercial, or not-for-profit sectors.

## Conflict of interest

The authors have no conflict of interest to declare.

## Ethical approval

This research was approved by Health Research Ethics Committee, University of Muhammadiyah Gombong in August 19, 2021 with the number 092.6/II.3.AU/F/KEPK/VIII/2021.

## Authors’ contribution

Each author contributed equally in all the parts of the research. All authors have critically reviewed and approved the final draft and are responsible for the content and similarity index of the manuscript.
